# BLNIMDA: identifying miRNA-disease associations based on weighted bi-level network

**DOI:** 10.1186/s12864-022-08908-8

**Published:** 2022-10-05

**Authors:** Junliang Shang, Yi Yang, Feng Li, Boxin Guan, Jin-Xing Liu, Yan Sun

**Affiliations:** School of Computer Science, Qufu Normal University, 276826 Rizhao, China

**Keywords:** miRNA similarity, Disease similarity, Association type, Bi-level network

## Abstract

**Background:**

MicroRNAs (miRNAs) have been confirmed to be inextricably linked to the emergence of human complex diseases. The identification of the disease-related miRNAs has gradually become a routine way to unveil the genetic mechanisms of examined disorders.

**Methods:**

In this study, a method BLNIMDA based on a weighted bi-level network was proposed for predicting hidden associations between miRNAs and diseases. For this purpose, the known associations between miRNAs and diseases as well as integrated similarities between miRNAs and diseases are mapped into a bi-level network. Based on the developed bi-level network, the miRNA-disease associations (MDAs) are defined as strong associations, potential associations and no associations. Then, each miRNA-disease pair (MDP) is assigned two information properties according to the bidirectional information distribution strategy, i.e., associations of miRNA towards disease and vice-versa. Finally, two affinity weights for each MDP obtained from the information properties and the association type are then averaged as the final association score of the MDP. Highlights of the BLNIMDA lie in the definition of MDA types, and the introduction of affinity weights evaluation from the bidirectional information distribution strategy and defined association types, which ensure the comprehensiveness and accuracy of the final prediction score of MDAs.

**Results:**

Five-fold cross-validation and leave-one-out cross-validation are used to evaluate the performance of the BLNIMDA. The results of the Area Under Curve show that the BLNIMDA has many advantages over the other seven selected computational methods. Furthermore, the case studies based on four common diseases and miRNAs prove that the BLNIMDA has good predictive performance.

**Conclusions:**

Therefore, the BLNIMDA is an effective method for predicting hidden MDAs.

**Supplementary Information:**

The online version contains supplementary material available at 10.1186/s12864-022-08908-8.

## Background

MicroRNAs (MiRNAs) are a type of non-protein-coding ribonucleic acids with a length of approximately 22 nucleotides [1]. It modulates the biological activities of proteins by promoting and inhibiting the expression of respective genes, thereby being able to initiate the emergence of diseases [2]. In recent years, miRNAs have been confirmed to be inextricably linked to the emergence of complex human diseases. For example, upregulation of miR-132 can contribute to the development of AIDS disease via promoting the replication of human immunodeficiency virus 1 (HIV-1) [3, 4]. Therefore, the identification of the hidden miRNA-disease associations (MDAs) can contribute to the better prevention and curation of diseases [5]. However, traditional “wet experiments” in biomedicine are often time-consuming and labor-intensive, characterizing by the lack of directionality to a certain extent [6, 7]. Therefore, it is necessary and meaningful to predict MDAs through biological methods. So far, many biological computation models for predicting MDAs have been developed [8–10], which can be divided into three main categories.

The first category of computational methods is represented by models constructed on the basis of score functions. The assumption that functionally similar miRNAs are more likely to be linked with similar diseases was implemented in the computational method for inference of MDAs devised by Jiang et al. [11]. This model firstly mapped the known MDAs, and the relationships between different miRNAs and diseases into a heterogeneous network to prove the correctness of the implemented hypothesis. Then, a scoring system for predicting MDAs was designed based on the neighbors shared by miRNAs and the measurements of their shortest paths. Therefore, the final MDA score was determined by ranking the disease-related miRNAs. In turns, Chen et al. [8] proposed a different model for predicting MDAs, called WBSMDA, which expanded the angle of miRNA and disease similarity calculation, innovatively employing the Gaussian interaction profile (GIP) kernel similarity network. It not only described the relationship between different miRNAs and diseases from a new perspective, but also could be used for independent nodes without any known associations.

The second category of computational methods for predicting MDAs is represented by models constructed on the basis of complex networks. These models mapped matrices related to miRNA and disease into the networks to predict the score of MDAs. Among those models, Chen et al. [12] proposed the RWRMDA method based on the random walk with restart to predict MDAs. The highlight of the RWRMDA was that the random walk was applied to the miRNA functional similarity network to rank all disease-related miRNAs. However, the RWRMDA ignored prior information and local topological structures of isolated miRNA and disease nodes. To eliminate the above defect, Xuan et al. [13] proposed a novel model based on the miRNA-related network constructed by integrating the available information on the MDAs and their local topological structures. The resulted nodes can be assigned as marked and unmarked whose characteristics enable the establishment of the transition network, which was proportional to the similarity between all nodes. For the isolated diseases, the bilayer network of MDAs was then constructed and random walks were extended to it. You et al. [9] constructed a multi-path heterogeneous network of MDAs (PBMDA), which integrated the known human MDAs, the miRNA-related similarity, the disease-related similarity, and the GIP kernel similarity. PBMDA set the length between different nodes on this network and further adopted a depth-first search algorithm to obtain the hidden MDAs information.

The third category of computational methods used for MDAs prediction is represented by models based on the machine learning algorithms. In this case, Chen et al. [14] proposed a matrix factorization model (IMCMDA), which solved the common problem of traditional methods, that is, the inability of isolated nodes to predict dependencies. The IMCMDA constructed features for miRNAs and diseases based on the information of the miRNA and disease similarity and selected robust features through an alternate search algorithm. Finally, the IMCMDA predicted the MDAs score through a semi-supervised model that did not rely on negative samples. In recent years, more and more deep learning-related models have been introduced into MDAs prediction. Ji et al. [15] proposed a computational framework based on deep autoencoder (AEMDA). The innovation of the AEMDA was the development of a learning-based feature extraction method after constructing miRNA and disease feature representation. Furthermore, the deep autoencoder and the reconstruction error method were introduced to predict MDAs. Tang et al. [16] developed a model (MMGCN) based on a multi-view multichannel attention graph convolutional network (GCN). The MMGCN employed the GCN to obtain features of miRNAs and diseases from multiple angles, and a multi-channel attention mechanism was used to adaptively select the important features. The final embeddings were constructed by a Convolutional Neural Networks synthesizer. The final association prediction was equated to the recommendation task, and a matrix completion was applied to predict hidden MDAs. For predicting MDAs from a comprehensive and novel perspective, Chu et al. [17] developed an original model (MDA-DCNFTG) based on the GCN, which treated MDAs prediction as a node classification task. The highlight of the MDA-GCNFTG was that it used graph sampling to predict MDAs from the perspective of feature and topological graphs based on Graph Convolutional Networks (GCNs). In addition, MDA-GCNFTG could predict not only new MDAs but also hidden association between diseases without known related miRNAs and miRNAs without known related diseases. Dai et al. [18] proposed a model for identifying potential MDAs based on the cascade forest model (MDA-CF), which integrated multi-source information to comprehensively characterize miRNAs and diseases, and used autoencoders for dimensionality reduction to obtain the optimal feature space and ranked it. A joint forest model was used in the prediction of potential MDAs.

In this study, we developed an MDAs prediction method based on a weighted bi-level network, named BLNIMDA. Specifically, a bi-level network is constructed based on the known MDAs as well as integrated similarities between miRNAs and diseases. In this constructed network, MDAs are defined into three categories, including strong associations, potential associations, and no associations. Then, each miRNA-disease pair (MDP) is assigned two information properties according to the bidirectional information distribution strategy. Finally, the association score of every MDP is obtained by averaging two affinity weights, which are derived from their information properties and association type. The Area Under Curve (AUC) values of five-fold cross-validation (FFCV) and leave-one-out cross-validation (LOOCV) are 0.9145 and 0.9176, respectively, which show that the BLNIMDA outperforms the other seven selected computational methods. Furthermore, the case studies based on four common diseases and miRNAs prove that the BLNIMDA has a good predictive performance. Thus, BLNIMDA can be used as a powerful tool to predict potential MDAs.

## Materials and methods

### Human MDAs

The data of MDAs are obtained from the HMDD v2.0 [19], which contains 5430 experimentally verified MDAs between 495 miRNAs and 383 diseases. To make better use of these information, we construct it as a matrix $A \in R^{{n_{m} \times n_{d} }}$, where $n_{d}$ and $n_{m}$ represent the number of diseases and miRNAs, respectively. In addition, if disease *g* and miRNA _*h*_ are confirmed to have an association, then $A\left( {g,h} \right)$ will be 1, otherwise 0.

### MiRNA function similarity

Wang et al. [20] developed a method for calculating the functional similarity of miRNAs, which was based on the assumption that similar miRNAs are more likely to be associated with similar diseases. The similarity score information of all miRNAs was obtained from http://www.cuilab.cn/files/images/cuilab/misim.zip. In this study, a $n_{m} * n_{m}$ matrix $MFS$ was constructed to indicate that miRNA similarity and the function similarity between miRNA *g* and miRNA _*h*_ can be expressed as $MFS\left( {g,h} \right)$.

### Disease semantic similarity frame *I*

**Fig. 1:**
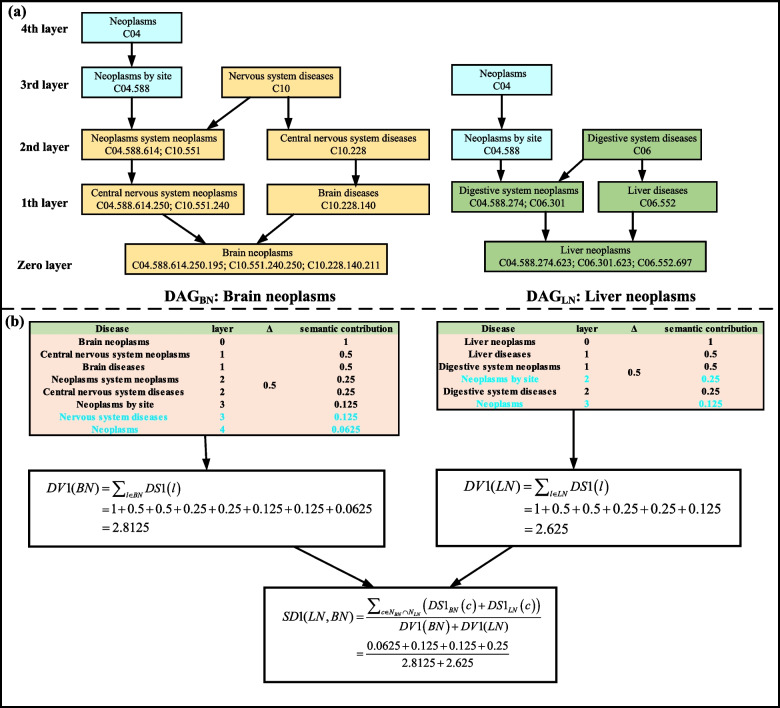
The computational process of the semantic similarity between Brain neoplasms and Liver neoplasms by Disease semantic similarity frame *I*

Disease semantic similarity is calculated from the hierarchical directed acyclic graph (DAG) of each disease [20]. The MeSH database (http://www.nlm.nih.gov/) contains the DAG information of all diseases. The semantic similarity scores between different diseases can be calculated by the relationship between their DAGs [19, 20]. Fig. 1 (a) shows the DAGs of brain neoplasms and liver neoplasms, where each node represents a specific disease MeSH descriptor. The DAG for the disease $Z$ can be denoted as $DAG_{Z} \left( {N_{Z} ,E_{Z} } \right)$, where $N_{Z}$ represents the node set, which includes the MeSH descriptor for disease $Z$ and its ancestor nodes, and $E_{Z}$ denotes the layer set, which includes all edges connecting the parent node to the child node $DAG_{Z}$. We assumed that $l$ is a MeSH descriptor node in $DAG_{Z}$, and its semantic contribution value in $DAG_{Z}$ is as follows:

1 $$\left\{ {\begin{array}{*{20}c} {DS{1}_{Z} \left( l \right) = {1 }if \, l = Z \, } \\ {DS{1}_{Z} \left( l \right) = \max \left\{ {\Delta * DS{1}\left( {l^{\prime}} \right)} \right\}\begin{array}{*{20}c} {} & {} \\ \end{array} if \, l \ne Z} \\ \end{array} } \right.$$

where $l^{\prime}$ is the child node of $l$ and $\Delta$ is used to indicate the semantic contribution decay, which is set to 0.5 according to previous literature [14] The semantic contribution of disease $Z$ is defined as formula (2).

2 $$DV{1}\left( Z \right) = \sum\nolimits_{{l \in N_{Z} }} {DS{1}\left( l \right)}$$

It can be found that the more shared MeSH descriptor nodes in the DAG of the two diseases, the more similar the two diseases are. Therefore, the formula for calculating the semantic similarity score of disease $d_{g}$ and $d_{h}$ is as follows:

3 $$SD{1}\left( {d_{g} ,d_{h} } \right) = \frac{{\sum\nolimits_{{c \in N_{{d_{g} }} \cap N_{{d_{h} }} }} {\left( {DS{1}_{{d_{g} }} \left( c \right) + DS{1}_{{d_{h} }} \left( c \right)} \right)} }}{{DV{1}\left( {d_{g} } \right) + DV{1}\left( {d_{h} } \right)}}$$

According to Eq. (3), $c$ denotes shared ancestral MeSH descriptor nodes by disease $d_{g}$ and $d_{h}$. The computational process of semantic similarity based on frame *I* for brain neoplasms and liver neoplasms is shown in Fig. 1 (b).

### Disease semantic similarity frame *II*

To differentiate the semantic contribution values of different diseases, which appear in the same layer of the same disease DAG, another calculation frame [21] was proposed. In this case the formula for calculating the semantic contribution value of disease $l$ is as follows:

4 $$DS{2}_{Z} \left( l \right) = - \log \left( {\frac{{N_{l} }}{N}} \right)$$

where $N_{l}$ represents the number of disease DAGs contained the MeSH descriptor of disease $l$ and $N$ denotes the number of all diseases in the MeSH database, i.e., $N_{l} /N$ is the probability that the MeSH descriptor of disease $l$ is present in all DAGs in the MeSH database. Based on frame *II*, the formula for calculating the semantic similarity score of disease $d_{g}$ and $d_{h}$ is as follows:

5 $$SD{2}\left( {d_{g} ,d_{h} } \right) = \frac{{\sum\nolimits_{{c \in N_{{d_{g} }} \cap N_{{d_{h} }} }} {\left( {DS{2}_{{d_{g} }} \left( c \right) + DS{2}_{{d_{h} }} \left( c \right)} \right)} }}{{DV{2}\left( {d_{g} } \right) + DV{2}\left( {d_{h} } \right)}}$$

where

6 $$DV{2}\left( Z \right) = \sum\nolimits_{{l \in N_{Z} }} {DS{2}\left( l \right)}$$

Finally, the similarity of different diseases at the semantic level is obtained by averaging the above two frames, which is shown as follows:

7 $$DS\left( {d_{g} ,d_{h} } \right) = \frac{{SD{1}\left( {d_{g} ,d_{h} } \right) + SD{2}\left( {d_{g} ,d_{h} } \right)}}{{2}}$$

### GIP kernel similarity

The GIP kernel [22] similarity aimed to measure the biological entities similarity based on their interaction profile information. GIP kernel similarity has been successfully introduced to the calculation of non-coding RNA and disease similarity [23]. In the adjacency matrix $A$, the $g$ row denotes the correlation vector between miRNA $g$ and 383 diseases, and the $h$ column indicates the correlation vector between disease $h$ and 495 miRNAs. We used $IP\left( {m_{g} } \right)$ and $IP\left( {d_{h} } \right)$ to represent them respectively. The formula for calculating the GIP kernel similarity of miRNAs and diseases is as follows:

8 $$KD\left( {d_{g} ,d_{h} } \right) = \exp \left( { - \beta_{d} ||IP\left( {d_{g} } \right) - IP\left( {d_{h} } \right)||^{{2}} } \right)$$

9 $$KM\left( {m_{g} ,m_{h} } \right) = \exp \left( { - \beta_{m} ||IP\left( {m_{g} } \right) - IP\left( {m_{h} } \right)||^{{2}} } \right)$$

where

10 $$\beta_{d} = \beta^{*}_{d} /\left( {\frac{{\sum\limits_{{h = {1}}}^{n} {||IP\left( {d_{h} } \right)||^{{2}} } }}{{n_{d} }}} \right)$$

11 $$\beta_{m} = \beta^{*}_{m} /\left( {\frac{{\sum\limits_{{g = {1}}}^{m} {||IP\left( {m_{g} } \right)||^{{2}} } }}{{n_{m} }}} \right)$$

The original bandwidth $\beta^{*}_{d}$ and $\beta^{*}_{m}$ is set to 1.0 [24, 25].

### Integrating similarity

Whether miRNA functional similarity, disease semantic similarity or GIP kernel similarity, they only provide a single aspect of similarity. Therefore, it is essential to integrate the above similarity information to obtain a more accurate and comprehensive disease or miRNA similarity. For example, if miRNA $m_{g}$ and miRNA $m_{h}$ have functional similarity, they will be retained, otherwise it will be equal to $KM\left( {m_{g} ,m_{h} } \right)$. Disease similarity is integrated using the same way. Therefore, the integration method is showed as follows:

12 $$FMS\left( {m_{g} ,m_{h} } \right) = \left\{ {\begin{array}{*{20}c} {MS\left( {m_{g} ,m_{h} } \right), \, m_{g} {\kern 1pt} and \, m_{h} \, have \, functional \, similarity} \\ {{\kern 1pt} {\kern 1pt} {\kern 1pt} {\kern 1pt} {\kern 1pt} {\kern 1pt} {\kern 1pt} {\kern 1pt} {\kern 1pt} {\kern 1pt} {\kern 1pt} {\kern 1pt} {\kern 1pt} {\kern 1pt} {\kern 1pt} {\kern 1pt} {\kern 1pt} {\kern 1pt} KM\left( {m_{g} ,m_{h} } \right), \, otherwise} \\ \end{array} {\kern 1pt} } \right.$$

13 $$FDS\left( {d_{g} ,d_{h} } \right) = \left\{ {\begin{array}{*{20}c} {DS\left( {d_{g} ,d_{h} } \right), \, d_{g} \, and \, d_{h} \, has \, semantic \, similarity} \\ {{\kern 1pt} {\kern 1pt} {\kern 1pt} {\kern 1pt} {\kern 1pt} {\kern 1pt} {\kern 1pt} {\kern 1pt} {\kern 1pt} {\kern 1pt} {\kern 1pt} {\kern 1pt} {\kern 1pt} {\kern 1pt} {\kern 1pt} {\kern 1pt} {\kern 1pt} {\kern 1pt} {\kern 1pt} KD\left( {d_{g} ,d_{h} } \right), \, otherwise} \\ \end{array} } \right.$$

An example of data processing, including the calculation of GIP kernel similarity and the process of integrating similarity, is shown in Figure S1 in the supplementary file.

### BLNIMDA

**Fig. 2:**
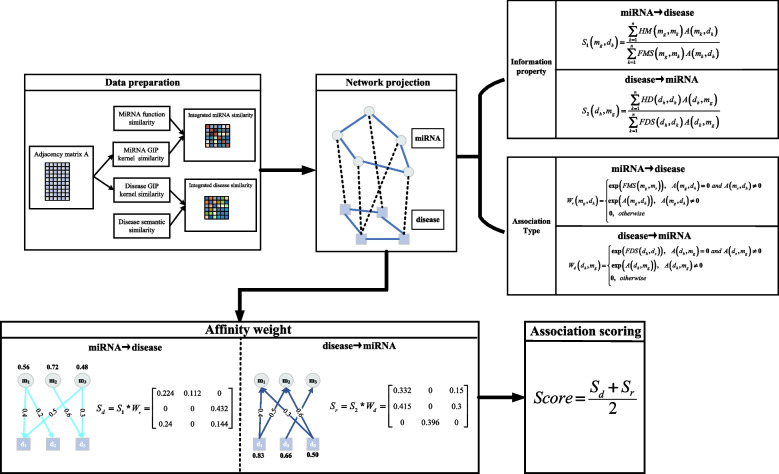
The flow chart of BLNIMDA

On the basis of the hypothesis that functionally similar miRNAs are more likely to be linked with similar diseases, we proposed a method named BLNIMDA that combines the above-processed data, including integrated miRNA similarity, integrated disease similarity and MDAs and these data are mapped into a bi-level weighted network. The BLNIMDA predicts the MDAs based on this network and Fig. 2 provides a detailed visualization of the BLNIMDA flow. Accordingly, the BLNIMDA integrates four main computational steps, including: (i) The determination of the miRNA function similarity, the disease semantic similarity and GIP kernel similarities; (ii) The integration of the estimated similarities and mapping MDAs into a bi-level weighted network; (iii) The generation of two side information properties for each MDAs through bidirectional information construction and assignment of all MDAs into three categories, namely strong associations, potential associations, and no associations. (iv) The estimation of two affinity weights for each MDP through bidirectional information construction strategy and its association type, and then averaged as the final MDAs score. Considering the direction of miRNAs to diseases as an example, the information properties of each MDP is defined as formula (14):

14 $$S_{1} \left( {m_{g} ,d_{h} } \right) = \frac{{\sum\limits_{{k = {1}}}^{n} {HM\left( {m_{g} ,m_{k} } \right)A\left( {m_{k} ,d_{h} } \right)} }}{{\sum\limits_{{k = {1}}}^{n} {FMS\left( {m_{g} ,m_{k} } \right)A\left( {m_{k} ,d_{h} } \right)} }}$$

where

15 $$HM\left( {m_{g} ,m_{k} } \right) = \left\{ {\begin{array}{*{20}c} {FMS\left( {m_{g} ,m_{k} } \right) \, FMS\left( {m_{g} ,m_{k} } \right) \ge T \, } \\ {{\kern 1pt} {\kern 1pt} {\kern 1pt} {\kern 1pt} {\kern 1pt} {\kern 1pt} {\kern 1pt} {\kern 1pt} {\kern 1pt} {\kern 1pt} {\kern 1pt} {\kern 1pt} {\kern 1pt} {\kern 1pt} {\kern 1pt} {\kern 1pt} {\kern 1pt} {\kern 1pt} {\kern 1pt} {\kern 1pt} {\kern 1pt} {\kern 1pt} {\kern 1pt} {\kern 1pt} {\kern 1pt} {0}{\kern 1pt} {\kern 1pt} {\kern 1pt} {\kern 1pt} {\kern 1pt} {\kern 1pt} {\kern 1pt} {\kern 1pt} {\kern 1pt} {\kern 1pt} {\kern 1pt} {\kern 1pt} {\kern 1pt} {\kern 1pt} {\kern 1pt} {\kern 1pt} {\kern 1pt} {\kern 1pt} {\kern 1pt} {\kern 1pt} {\kern 1pt} {\kern 1pt} {\kern 1pt} {\kern 1pt} {\kern 1pt} {\kern 1pt} {\kern 1pt} {\kern 1pt} {\kern 1pt} {\kern 1pt} {\kern 1pt} {\kern 1pt} {\kern 1pt} {\kern 1pt} {\kern 1pt} {\kern 1pt} {\kern 1pt} {\kern 1pt} {\kern 1pt} {\kern 1pt} {\kern 1pt} {\kern 1pt} {\kern 1pt} {\kern 1pt} {\kern 1pt} {\kern 1pt} {\kern 1pt} {\kern 1pt} {\kern 1pt} {\kern 1pt} {\kern 1pt} {\kern 1pt} {\kern 1pt} {\kern 1pt} {\kern 1pt} {\kern 1pt} {\kern 1pt} {\kern 1pt} {\kern 1pt} {\kern 1pt} {\kern 1pt} {\kern 1pt} {\kern 1pt} {\kern 1pt} \, otherwise} \\ \end{array} } \right.$$

Considering that the weak similarity nodes of $m_{g}$ may affect the accuracy of prediction results, we set the parameter $T$ to remove weak similar nodes. The MDAs are defined into three types: (i) $m_{g}$ and $d_{h}$ have strong association when they display unequivocal reciprocal association; (ii) $m_{g}$ and $d_{h}$ have potential association when they do not display direct association but the most similar node $m_{s}$ to $m_{g}$ has an unequivocal association with $d_{h}$. (iii) otherwise, there is not any potential association. Three types of MDAs are shown in the formula (16).

16 $$W_{r} \left( {m_{g} ,d_{h} } \right) = \left\{ {\begin{array}{*{20}c} {\exp \left( {FMS\left( {m_{g} ,m_{s} } \right)} \right){\kern 1pt} {\kern 1pt} {\kern 1pt} {\kern 1pt} {\kern 1pt} {\kern 1pt} {\kern 1pt} {\kern 1pt} {\kern 1pt} A\left( {m_{g} ,d_{h} } \right) = {0}{\kern 1pt} {\kern 1pt} {\kern 1pt} {\kern 1pt} {\kern 1pt} and{\kern 1pt} {\kern 1pt} {\kern 1pt} {\kern 1pt} {\kern 1pt} A\left( {m_{s} ,d_{h} } \right) \ne {0}} \\ {\exp \left( {A\left( {m_{g} ,d_{h} } \right)} \right){\kern 1pt} {\kern 1pt} {\kern 1pt} {\kern 1pt} {\kern 1pt} {\kern 1pt} {\kern 1pt} {\kern 1pt} {\kern 1pt} {\kern 1pt} {\kern 1pt} {\kern 1pt} {\kern 1pt} {\kern 1pt} {\kern 1pt} {\kern 1pt} {\kern 1pt} {\kern 1pt} {\kern 1pt} {\kern 1pt} {\kern 1pt} A\left( {m_{g} ,d_{h} } \right) \ne {0}{\kern 1pt} {\kern 1pt} {\kern 1pt} {\kern 1pt} {\kern 1pt} {\kern 1pt} {\kern 1pt} {\kern 1pt} {\kern 1pt} {\kern 1pt} {\kern 1pt} {\kern 1pt} {\kern 1pt} {\kern 1pt} {\kern 1pt} {\kern 1pt} {\kern 1pt} {\kern 1pt} {\kern 1pt} {\kern 1pt} {\kern 1pt} {\kern 1pt} {\kern 1pt} {\kern 1pt} {\kern 1pt} {\kern 1pt} {\kern 1pt} {\kern 1pt} {\kern 1pt} {\kern 1pt} {\kern 1pt} {\kern 1pt} {\kern 1pt} {\kern 1pt} {\kern 1pt} {\kern 1pt} {\kern 1pt} {\kern 1pt} {\kern 1pt} {\kern 1pt} {\kern 1pt} {\kern 1pt} {\kern 1pt} {\kern 1pt} {\kern 1pt} {\kern 1pt} {\kern 1pt} {\kern 1pt} {\kern 1pt} {\kern 1pt} {\kern 1pt} {\kern 1pt} {\kern 1pt} {\kern 1pt} {\kern 1pt} {\kern 1pt} {\kern 1pt} {\kern 1pt} {\kern 1pt} {\kern 1pt} {\kern 1pt} {\kern 1pt} {\kern 1pt} {\kern 1pt} {\kern 1pt} {\kern 1pt} {\kern 1pt} {\kern 1pt} {\kern 1pt} {\kern 1pt} {\kern 1pt} {\kern 1pt} {\kern 1pt} {\kern 1pt} {\kern 1pt} {\kern 1pt} {\kern 1pt} {\kern 1pt} {\kern 1pt} {\kern 1pt} {\kern 1pt} } \\ {{0}{\kern 1pt} {\kern 1pt} {\kern 1pt} {\kern 1pt} {\kern 1pt} {\kern 1pt} {\kern 1pt} {\kern 1pt} {\kern 1pt} {\kern 1pt} {\kern 1pt} {\kern 1pt} {\kern 1pt} {\kern 1pt} {\kern 1pt} {\kern 1pt} {\kern 1pt} {\kern 1pt} {\kern 1pt} {\kern 1pt} {\kern 1pt} {\kern 1pt} {\kern 1pt} {\kern 1pt} {\kern 1pt} {\kern 1pt} {\kern 1pt} {\kern 1pt} {\kern 1pt} {\kern 1pt} {\kern 1pt} {\kern 1pt} {\kern 1pt} {\kern 1pt} {\kern 1pt} {\kern 1pt} {\kern 1pt} {\kern 1pt} {\kern 1pt} {\kern 1pt} {\kern 1pt} {\kern 1pt} {\kern 1pt} {\kern 1pt} {\kern 1pt} {\kern 1pt} {\kern 1pt} {\kern 1pt} {\kern 1pt} {\kern 1pt} {\kern 1pt} {\kern 1pt} {\kern 1pt} {\kern 1pt} otherwise{\kern 1pt} {\kern 1pt} {\kern 1pt} {\kern 1pt} {\kern 1pt} {\kern 1pt} {\kern 1pt} {\kern 1pt} {\kern 1pt} {\kern 1pt} {\kern 1pt} {\kern 1pt} {\kern 1pt} {\kern 1pt} {\kern 1pt} {\kern 1pt} {\kern 1pt} {\kern 1pt} {\kern 1pt} {\kern 1pt} {\kern 1pt} {\kern 1pt} {\kern 1pt} {\kern 1pt} {\kern 1pt} {\kern 1pt} {\kern 1pt} {\kern 1pt} {\kern 1pt} {\kern 1pt} {\kern 1pt} {\kern 1pt} {\kern 1pt} {\kern 1pt} {\kern 1pt} {\kern 1pt} {\kern 1pt} {\kern 1pt} {\kern 1pt} {\kern 1pt} {\kern 1pt} {\kern 1pt} {\kern 1pt} {\kern 1pt} {\kern 1pt} {\kern 1pt} {\kern 1pt} {\kern 1pt} {\kern 1pt} {\kern 1pt} {\kern 1pt} {\kern 1pt} {\kern 1pt} {\kern 1pt} {\kern 1pt} {\kern 1pt} {\kern 1pt} {\kern 1pt} {\kern 1pt} {\kern 1pt} {\kern 1pt} {\kern 1pt} {\kern 1pt} {\kern 1pt} {\kern 1pt} {\kern 1pt} } \\ \end{array} } \right.$$

where $m_{s}$ is the miRNA with the greatest similarity to $m_{g}$. From miRNA to disease, the affinity weight of each MDP are defined through a bidirectional information construction strategy and its association type according to formula (17):

17 $$S_{d} = S_{{1}} *W_{r}$$

From disease to miRNA direction, the information property of each MDP is the same as the above steps, and the details are as follows:

18 $$S_{2} \left( {d_{h} ,m_{g} } \right) = \frac{{\sum\limits_{k = 1}^{n} {HD\left( {d_{h} ,d_{k} } \right)A\left( {d_{k} ,m_{g} } \right)} }}{{\sum\limits_{k = 1}^{n} {FDS\left( {d_{h} ,d_{k} } \right)A\left( {d_{k} ,m_{g} } \right)} }}$$

where

19 $$HD\left( {d_{h} ,d_{k} } \right) = \left\{ {\begin{array}{*{20}c} {FDS\left( {d_{h} ,d_{k} } \right) \, FDS\left( {d_{h} ,d_{k} } \right) \ge T} \\ {{0}{\kern 1pt} {\kern 1pt} {\kern 1pt} {\kern 1pt} {\kern 1pt} {\kern 1pt} {\kern 1pt} {\kern 1pt} {\kern 1pt} {\kern 1pt} {\kern 1pt} {\kern 1pt} {\kern 1pt} {\kern 1pt} {\kern 1pt} {\kern 1pt} {\kern 1pt} {\kern 1pt} {\kern 1pt} {\kern 1pt} {\kern 1pt} {\kern 1pt} {\kern 1pt} {\kern 1pt} {\kern 1pt} {\kern 1pt} {\kern 1pt} \, {\kern 1pt} {\kern 1pt} {\kern 1pt} {\kern 1pt} {\kern 1pt} {\kern 1pt} otherwise} \\ \end{array} } \right.$$

The three types of miRNA-disease association are as follows:

20 $$W_{d} \left( {d_{h} ,m_{g} } \right) = \left\{ {\begin{array}{*{20}c} {\exp \left( {FDS\left( {d_{h} ,d_{s} } \right)} \right){\kern 1pt} {\kern 1pt} {\kern 1pt} {\kern 1pt} {\kern 1pt} {\kern 1pt} {\kern 1pt} {\kern 1pt} {\kern 1pt} A\left( {d_{h} ,m_{g} } \right) = {0}{\kern 1pt} {\kern 1pt} {\kern 1pt} {\kern 1pt} {\kern 1pt} and{\kern 1pt} {\kern 1pt} {\kern 1pt} {\kern 1pt} {\kern 1pt} A\left( {d_{s} ,m_{g} } \right) \ne {0}} \\ {\exp \left( {A\left( {d_{h} ,m_{g} } \right)} \right){\kern 1pt} {\kern 1pt} {\kern 1pt} {\kern 1pt} {\kern 1pt} {\kern 1pt} {\kern 1pt} {\kern 1pt} {\kern 1pt} {\kern 1pt} {\kern 1pt} {\kern 1pt} {\kern 1pt} {\kern 1pt} {\kern 1pt} {\kern 1pt} {\kern 1pt} {\kern 1pt} {\kern 1pt} {\kern 1pt} {\kern 1pt} A\left( {d_{h} ,m_{g} } \right) \ne {0}{\kern 1pt} {\kern 1pt} {\kern 1pt} {\kern 1pt} {\kern 1pt} {\kern 1pt} {\kern 1pt} {\kern 1pt} {\kern 1pt} {\kern 1pt} {\kern 1pt} {\kern 1pt} {\kern 1pt} {\kern 1pt} {\kern 1pt} {\kern 1pt} {\kern 1pt} {\kern 1pt} {\kern 1pt} {\kern 1pt} {\kern 1pt} {\kern 1pt} {\kern 1pt} {\kern 1pt} {\kern 1pt} {\kern 1pt} {\kern 1pt} {\kern 1pt} {\kern 1pt} {\kern 1pt} {\kern 1pt} {\kern 1pt} {\kern 1pt} {\kern 1pt} {\kern 1pt} {\kern 1pt} {\kern 1pt} {\kern 1pt} {\kern 1pt} {\kern 1pt} {\kern 1pt} {\kern 1pt} {\kern 1pt} {\kern 1pt} {\kern 1pt} {\kern 1pt} {\kern 1pt} {\kern 1pt} {\kern 1pt} {\kern 1pt} {\kern 1pt} {\kern 1pt} {\kern 1pt} {\kern 1pt} {\kern 1pt} {\kern 1pt} {\kern 1pt} {\kern 1pt} {\kern 1pt} {\kern 1pt} {\kern 1pt} {\kern 1pt} {\kern 1pt} {\kern 1pt} {\kern 1pt} {\kern 1pt} {\kern 1pt} {\kern 1pt} {\kern 1pt} {\kern 1pt} {\kern 1pt} {\kern 1pt} {\kern 1pt} {\kern 1pt} {\kern 1pt} {\kern 1pt} {\kern 1pt} {\kern 1pt} {\kern 1pt} {\kern 1pt} {\kern 1pt} } \\ {{0}{\kern 1pt} {\kern 1pt} {\kern 1pt} {\kern 1pt} {\kern 1pt} {\kern 1pt} {\kern 1pt} {\kern 1pt} {\kern 1pt} {\kern 1pt} {\kern 1pt} {\kern 1pt} {\kern 1pt} {\kern 1pt} {\kern 1pt} {\kern 1pt} {\kern 1pt} {\kern 1pt} {\kern 1pt} {\kern 1pt} {\kern 1pt} {\kern 1pt} {\kern 1pt} {\kern 1pt} {\kern 1pt} {\kern 1pt} {\kern 1pt} {\kern 1pt} {\kern 1pt} {\kern 1pt} {\kern 1pt} {\kern 1pt} {\kern 1pt} {\kern 1pt} {\kern 1pt} {\kern 1pt} {\kern 1pt} {\kern 1pt} {\kern 1pt} {\kern 1pt} {\kern 1pt} {\kern 1pt} {\kern 1pt} {\kern 1pt} {\kern 1pt} {\kern 1pt} {\kern 1pt} {\kern 1pt} {\kern 1pt} {\kern 1pt} {\kern 1pt} {\kern 1pt} {\kern 1pt} {\kern 1pt} otherwise{\kern 1pt} {\kern 1pt} {\kern 1pt} {\kern 1pt} {\kern 1pt} {\kern 1pt} {\kern 1pt} {\kern 1pt} {\kern 1pt} {\kern 1pt} {\kern 1pt} {\kern 1pt} {\kern 1pt} {\kern 1pt} {\kern 1pt} {\kern 1pt} {\kern 1pt} {\kern 1pt} {\kern 1pt} {\kern 1pt} {\kern 1pt} {\kern 1pt} {\kern 1pt} {\kern 1pt} {\kern 1pt} {\kern 1pt} {\kern 1pt} {\kern 1pt} {\kern 1pt} {\kern 1pt} {\kern 1pt} {\kern 1pt} {\kern 1pt} {\kern 1pt} {\kern 1pt} {\kern 1pt} {\kern 1pt} {\kern 1pt} {\kern 1pt} {\kern 1pt} {\kern 1pt} {\kern 1pt} {\kern 1pt} {\kern 1pt} {\kern 1pt} {\kern 1pt} {\kern 1pt} {\kern 1pt} {\kern 1pt} {\kern 1pt} {\kern 1pt} {\kern 1pt} {\kern 1pt} {\kern 1pt} {\kern 1pt} {\kern 1pt} {\kern 1pt} {\kern 1pt} {\kern 1pt} {\kern 1pt} {\kern 1pt} {\kern 1pt} {\kern 1pt} {\kern 1pt} {\kern 1pt} {\kern 1pt} } \\ \end{array} } \right.$$

In the same way, from disease to miRNA, the affinity weight of each MDP are determined through information property and the MDA type. The specific definition is as follows:

21 $$S_{r} = S_{2} *W_{d}$$

According to formulas (14)- (21), two affinity weights for each MDP are determined, and then averaged them as the final MDAs score following the formula (22):

22 $$S_{f} = \frac{{S_{d} + S_{r} }}{{2}}$$

A BLNIMDA calculation example, including the generation of two side information properties, the calculation of two affinity weights for each MDP and the MDA score, is shown in Figure S2 in the supplementary file.

## Results

### Performance evaluation

To validate the prediction performance of the BLNIMDA, we employed cross-validation, which is considered as a reasonably comprehensive measure. In this study, FFCV and LOOCV were used to verify the performance of the BLNIMDA. For the FFCV, all MDAs were randomly divided into five groups, and each group was chosen as the test sample set in turns. The remaining groups were used as the training sample sets. For LOOCV, each experimentally verified MDP was regarded in turn as the test samples where the rest of them were used as the training sample sets. The AUC and ROC were used to visualize the accuracy of the BLNIMDA in MDAs prediction. Specifically, the values closer to 1 indicated better performance of the BLNIMDA, on the contrary, the lower values of AUC revealed the worse prediction performance. By setting different thresholds, the ratio of samples above the threshold to other samples in all positive samples was taken as sensitivity (TPR), and the ratio of samples below the threshold to other samples in all negative samples was taken as 1-specificity (FPR). ROC curves were plotted by TPR and FPR under different thresholds.

### Effects of parameters

**Fig. 3:**
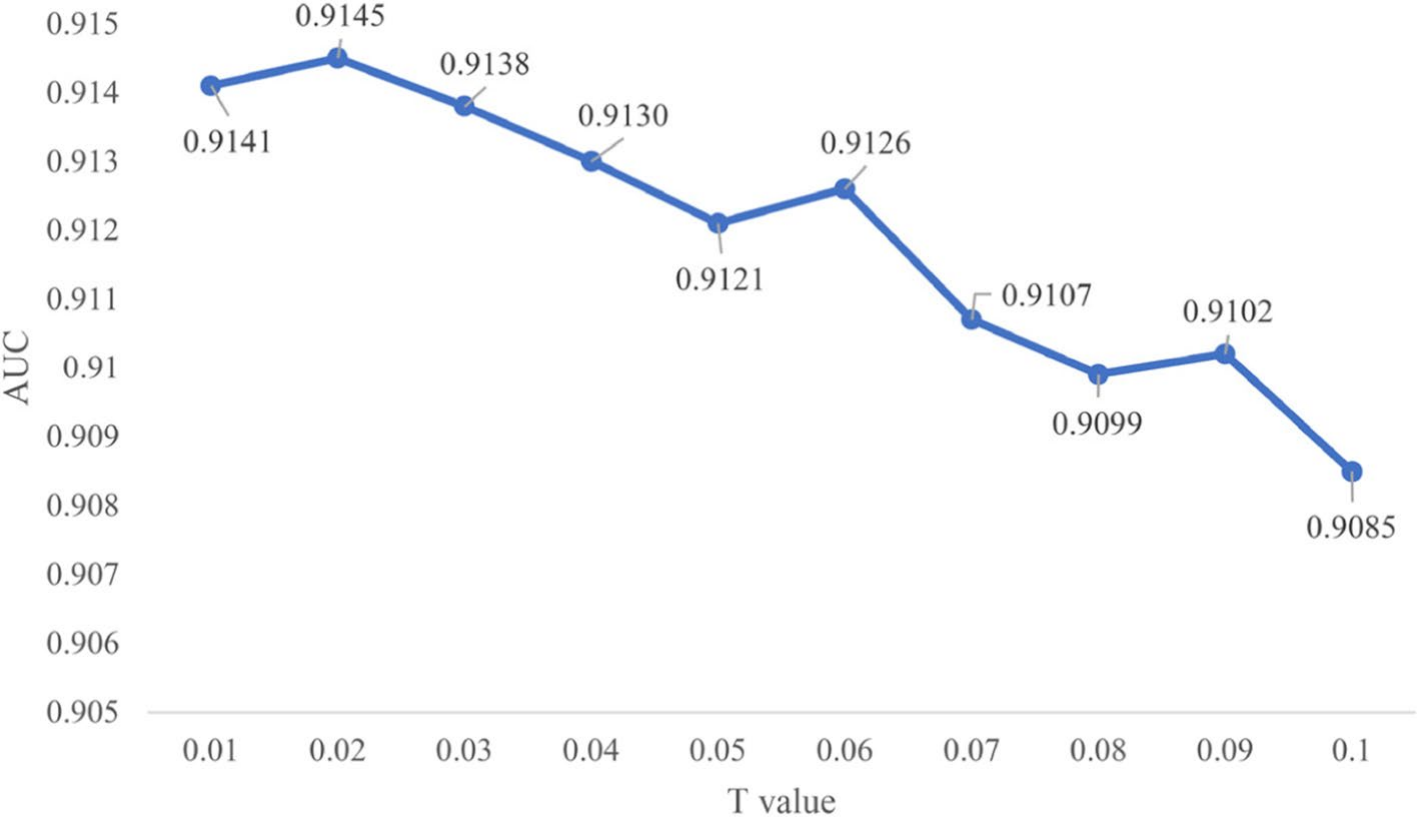
AUC values of BLNIMDA depending on the different $T$ parameter values

In the bidirectional information construction strategy, there are some noise nodes in the miRNA or disease similarity network, which may affect the performance of the BLNIMDA. In view of this situation, we set the parameter $T$ and adjusted the threshold of $T$ to reduce the influence of such nodes, so as to obtain the optimal prediction results. After several experiments, the comparison of the average AUC values after 100 times FFCV when $T$ sets different thresholds is shown in Fig. 3. It can be seen that the performance of the BLNIMDA is the best when $T$ is set to 0.02.

### Performance comparison

**Fig. 4:**
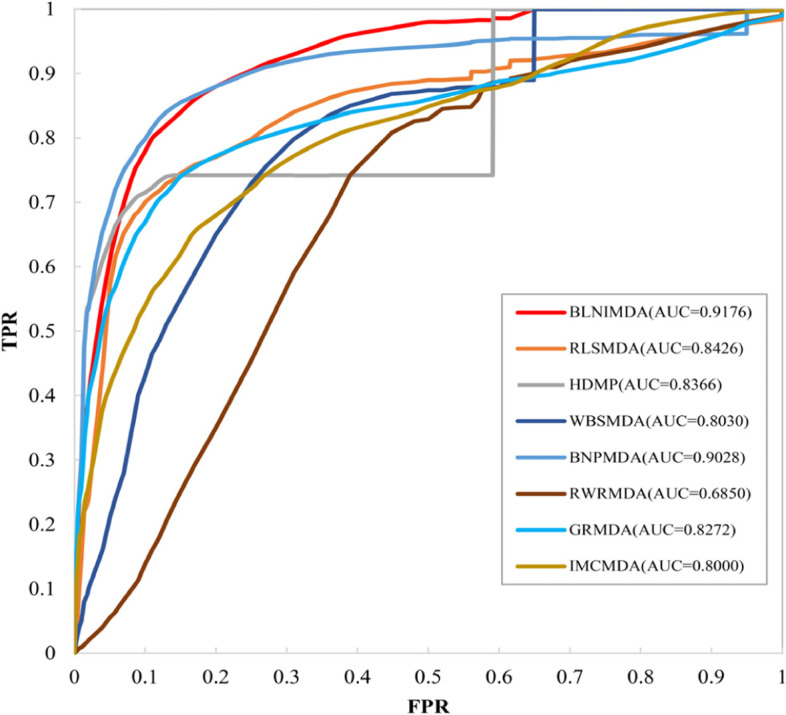
ROC curves and AUC values of compared methods in terms of LOOCV

**Fig. 5:**
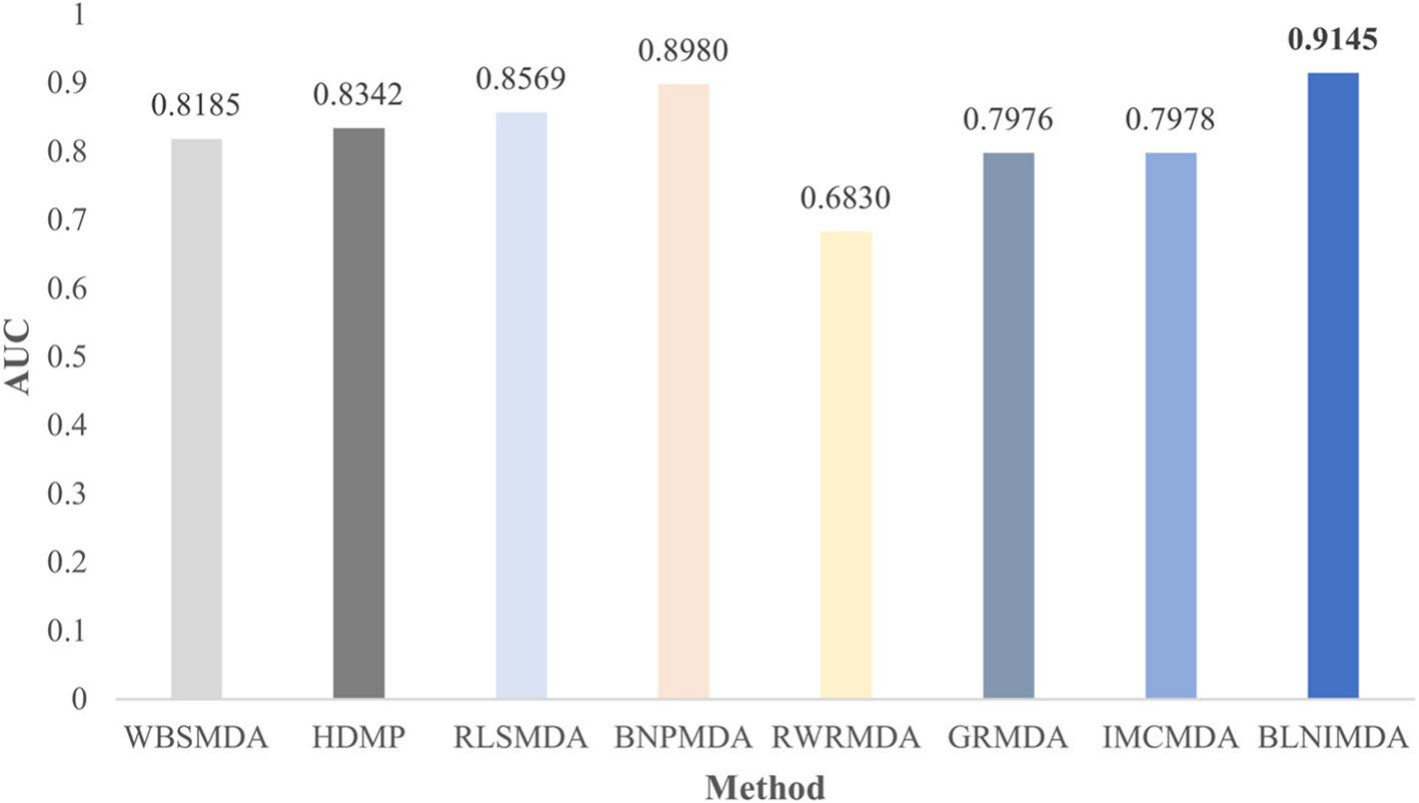
AUC values of compared computational methods for MDAs detection in terms of FFCV

In order to show the superiority of the BLNIMDA, we compared seven models [8, 10, 12, 14, 24, 26, 27] for predicting the MDAs. These seven models have their characteristics. The WBSMDA and the HDMP are basic models in the research field of predicting MDAs, and the RWRMDA is a restart random walk model, the GRMDA is a graph regression method, the RLSMDA is a model based on machine learning methods, the IMCMDA is a model completed by inductive matrix, and the BNPMDA is a high-level paper proposed in recent years. All models selected for comparative analysis, including the BLNIMDA, used the same network to evaluate the performance through FFCV and LOOCV. The ROC curves of the LOOCV are shown in Fig. 4. It can be clearly found that the AUC values of WBSMDA, HDMP, RWRMDA, GRMDA, RLSMDA, IMCMDA, and BNPMDA models are 0.8030, 0.8366, 0.6850, 0.8272, 0.8426, 0.8000, 0.9028, respectively. The AUC value of the BLNIMDA is 0.9176, which is higher than the other seven models. The results of the FFCV are shown in Fig. 5. The AUC value of the BLNIMDA model is 0.9145, while the AUC values of WBSMDA, HDMP, RLSMDA, BNPMDA, RWRMDA, GRMDA and IMCMDA models are 0.8185, 0.8342, 0.8569, 0.8980, 0.6830, 0.7976 and 0.7978, respectively. All seven models are lower than the BLNIMDA. Compared with the classical graph algorithm, our BLNIMDA model introduces affinity weights evaluation and the bidirectional information distribution strategy. In particular, it calculates the association score of miRNA and disease pair through the affinity weights evaluation from the bidirectional information distribution strategy and the introduced association types.

In addition, an independent dataset was used as the test set to better verify the prediction performance of BLNIMDA. Considering that BLNIMDA uses HMDD v2.0 as its training set, the MDAs in the new version of the HMDD database (HMDD v3.2) not included in the training dataset were used as a test sample. The AUCs of BLNIMDA and the other seven methods are shown in Table 1, from which it can be seen that the prediction performance of BLNIMDA in the independent test set is better than other comparison methods.

**Table 1:** AUC values of compared computational methods for MDAs detection in independent test set

**Method**	**AUC**
WBSMDA	0.7932
HDMP	0.8035
RLSMDA	0.8326
BNPMDA	0.8869
RWRMDA	0.6324
GRMDA	0.7574
IMCMDA	0.7658
**BLNIMDA**	**0.9032**

### Case studies

To verify the accuracy of the BLNIMDA, we employ it to predict the association between miRNAs and four important diseases, including lung neoplasms, breast neoplasms, gastric neoplasms, and colon neoplasms.

Colon neoplasms is one of the most common malignant neoplasms that seriously threaten human health, and its mortality rate ranks third in the world [25]. According to available statistics, the number of new cases of colon neoplasms worldwide in 2008 reached 1 million, half of which led to death [28]. In recent years, the number of new cases of colon neoplasms in China has been on the rise, which can be attributed to the improvement of their diagnosis effectiveness by the advancement of endoscopy and the higher frequency of their emergence caused by changes in dietary patterns. Therefore, colon cancer has become one of the most common malignant tumors, which seriously threatens human health [29, 30]. Lungcancer is another most common malignant tumors in the world. According to incomplete statistics, about 200,000 people are diagnosed with lung cancer every year [31]. Generally speaking, patients with lung cancer have no typical clinical manifestations in the early stage, and have already been in the advanced stage at the time of diagnosis, with a poor prognosis and the 5-year survival rate of less than 15% [32]. If lung cancer can be detected early and intervened, the survival rate of patients can be significantly increased [33]. Therefore, it is of great significance to find an effective early diagnostic marker. Breast neoplasm is another one of the most frequent malignancies and occurs in breast epithelial tissue in women [34]. Approximately 1.2 million women around the world are diagnosed each year. Gastric cancer is a particularly intractable malignant tumor with the fifth highest incidence and the third highest mortality in the world highly recurrent malignant tumors, after lung cancer and colorectal [35]. Gastric cancer is difficult to detect in the early stage and has no obvious characteristics, so many patients are diagnosed in advanced stage and the mortality rate is extremely high [35]. After scoring and ranking the MDAs by the BLNIMDA, all known miRNAs related to these four diseases were removed, and the remaining information was queried and verified in the HMDDv3.2 [36], the miRCancer [37] database and related literature. According to the results shown in Table 2, it can be seen that the 20 MDPs predicted by the BLNIMDA are all confirmed from other databases.

**Table 2:** The top five miRNAs identified by BLNIMDA to be associated with four important diseases

**Disease**	**Rank**	**miRNA**	**Evidence**	**Causality**
Lung Neoplasms	1	hsa-mir-21	miRCancer; HMDDv3.2	YES
	2	hsa-mir-155	miRCancer; HMDDv3.2	YES
	3	hsa-mir-146a	miRCancer; HMDDv3.2	NO
	4	hsa-mir-17	miRCancer; HMDDv3.2	YES
	5	hsa-mir-34a	miRCancer; HMDDv3.2	YES
Breast Neoplasms	1	hsa-mir-21	miRCancer; HMDDv3.2	YES
	2	hsa-mir-155	miRCancer; HMDDv3.2	YES
	3	hsa-mir-17	miRCancer; HMDDv3.2	YES
	4	hsa-mir-146a	miRCancer; HMDDv3.2	YES
	5	hsa-mir-34a	miRCancer; HMDDv3.2	YES
Gastric Neoplasms	1	hsa-mir-148a	miRCancer; HMDDv3.2	YES
	2	hsa-mir-23a	miRCancer; HMDDv3.2	YES
	3	hsa-mir-370	miRCancer; HMDDv3.2	YES
	4	hsa-mir-429	miRCancer; HMDDv3.2	YES
	5	hsa-mir-21	miRCancer; HMDDv3.2	YES
Colon Neoplasms	1	hsa-mir-145	miRCancer; HMDDv3.2	YES
	2	hsa-mir-17	HMDDv3.2	YES
	3	hsa-mir-21	miRCancer; HMDDv3.2	YES
	4	hsa-mir-126	miRCancer; HMDDv3.2	YES
	5	hsa-mir-155	miRCancer; HMDDv3.2	YES

At the same time, we randomly selected four miRNAs and screened the top five diseases with their association scores. The predicted MDAs were verified in HMDDv3.2 and miRCancer databases, and almost all of them were confirmed by available evidence data and shown in Table 3. Furthermore, causality information for the top five associations was verified by HMDD v3.2 and attached to Tables 2 and 3.

**Table 3:** The top five diseases identified by BLNIMDA to be associated with four major miRNAs

**MiRNA**	**Rank**	**Disease**	**Evidence**	**Causality**
hsa-mir-125a	1	Carcinoma	HMDDv3.2	YES
	2	Breast Neoplasms	miRCancer; HMDDv3.2	YES
	3	Lung Neoplasms	miRCancer; HMDDv3.2	YES
	4	Stomach Neoplasms	HMDDv3.2	NO
	5	Ovarian Neoplasms	miRCancer; HMDDv3.2	YES
hsa-mir-15a	1	Carcinoma, Hepatocellular	HMDDv3.2	YES
	2	Breast Neoplasms	miRCancer; HMDDv3.2	YES
	3	Melanoma	miRCancer; HMDDv3.2	YES
	4	Lung Neoplasms	HMDDv3.2	YES
	5	Colorectal Neoplasms	miRCancer; HMDDv3.2	NO
hsa-mir-150	1	Carcinoma, Hepatocellular	HMDDv3.2	YES
	2	Colorectal Neoplasms	HMDDv3.2	YES
	3	Lung Neoplasms	HMDDv3.2	YES
	4	Stomach Neoplasms	HMDDv3.2	NO
	5	Breast Neoplasms	HMDDv3.2	YES
hsa-mir-137	1	Lung Neoplasms	HMDDv3.2	YES
	2	Breast Neoplasms	HMDDv3.2	YES
	3	Carcinoma, Hepatocellular	miRCancer; HMDDv3.2	YES
	4	Stomach Neoplasms	miRCancer; HMDDv3.2	NO
	5	Melanoma	miRCancer; HMDDv3.2	YES

## Discussion

Many miRNAs have been confirmed to have links with the occurrence of human diseases. Discovering the potential MDAs is of great significance for understanding the pathogenesis of diseases. Traditional methods for finding MDAs have some disadvantages such as low efficiency and difficult operation. Therefore, many computational models have been proposed, which are mainly divided into three categories, namely score function-based, complex network-based and machine learning-based. In this study, firstly, we proposed a computational method BLNIMDA based on a weighted bi-level network to predict the hidden MDAs. Specifically, the bi-level network is constructed based on the known MDAs as well as integrated similarities between miRNAs and diseases, in which nodes denote miRNAs and diseases. In this developed bi-level network, the MDAs are defined as strong associations, potential associations and no association according to the relationship between the miRNAs and diseases. Then, each MDP is assigned two information properties based on the bidirectional information distribution strategy. Finally, two affinity weights for each MDP are obtained from the information properties and the association type and then averaged as the final association score of every MDP. In experiments, FFCV and LOOCV are used to evaluate the performance of the BLNIMDA, and the AUC values of them are 0.9145 and 0.9176, respectively, which show that the BLNIMDA have advantages over the other seven computational methods. Furthermore, the case studies based on four common diseases and miRNAs prove that the BLNIMDA has good predictive performance. Therefore, BLNIMDA is an effective method for predicting hidden MDAs.

The main contributions of the BLNIMDA are the ability for determining MDA type, and the introduction of two affinity weights in accordance to the bidirectional information distribution strategy and defined association types, which ensures the comprehensiveness and accuracy of the final prediction score for each MDP. However, BLNIMDA still has certain defects. First of all, the dependence on known information has not been completely eliminated. In addition, for the information between miRNAs and diseases, multiple dimensions similarity could be considered to improve prediction performance. Furthermore, BLNIMDA is essentially a traditional network method, which still needs to be improved through algorithms based on deep learning.

In the future, we will consider in-depth researches for MDAs, such as the regulatory role of miRNAs in specific diseases and the multiple type MDAs instead of taking them as binary, which have important implications for the treatment of complex human diseases.

## Conclusion

In this study, a method BLNIMDA based on a weighted bi-level network was proposed for predicting the hidden associations between miRNAs and diseases. Highlights of the BLNIMDA lie in the definition of MDA types, and the introduction of two affinity weights by the bidirectional information distribution strategy. FFCV, LOOCV and case studies based on four common diseases and miRNAs are used to evaluate the performance of the BLNIMDA. The experimental results show that BLNIMDA has achieved excellent prediction performance. However, the BLNIMDA still has room for improvement, such as integrating more comprehensive data on the miRNA and disease similarities.

## Supplementary Information

**Additional file 1: Figure S1.** An example of data processing, including the calculation of GIP kernel similarity and the process of integrating similarity. **Figure S2.** A BLNIMDA calculation example, including the generation of two side information properties, the calculation of two affinity weights for each MDP and the MDA score.

## Data Availability

The BLNIMDA is implemented in Matlab. Its source code, user manual and related experimental data are available online at https://github.com/CDMB-lab/BLNIMDA.
