# Supplementary material for: BLNIMDA: identifying miRNA-disease associations based on weighted bi-level network
Source: BMC Genomics. 2022 Oct 5;23:686. doi: 10.1186/s12864-022-08908-8 (PMC9533620; doi:10.1186/s12864-022-08908-8)
Supplement: Supplementary file 1 — Additional file 1: Figure S1. An example of data processing, including the calculation of GIP kernel similarity and the process of integrating similarity. Figure S2. A BLNIMDA calculation example, including the generation of two side information properties, the calculation of two affinity weights for each MDP and the MDA score. [file 12864_2022_8908_MOESM1_ESM.docx]

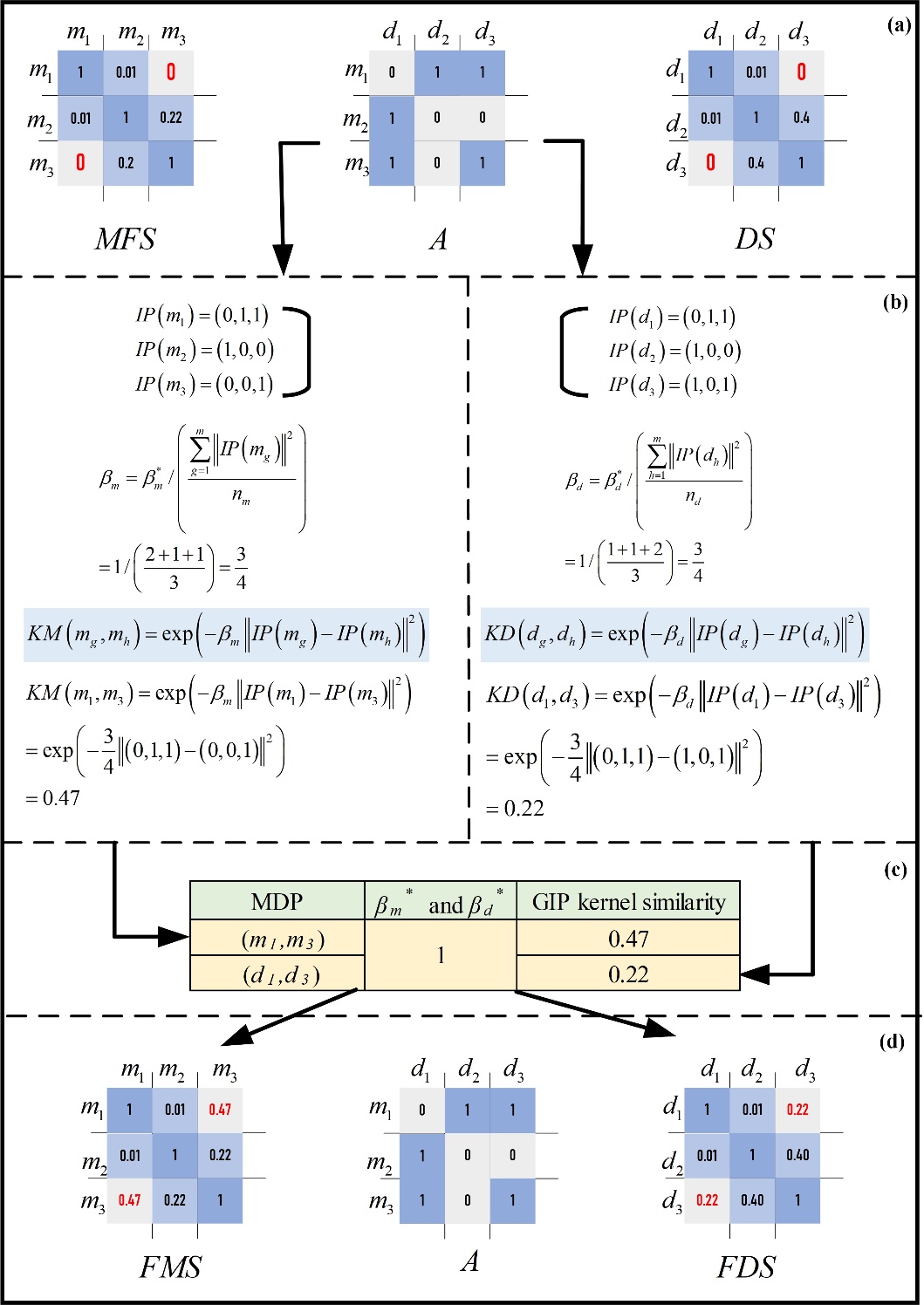


Figure S1. An example of data processing, including the calculation of GIP kernel similarity and the process of integrating similarity.


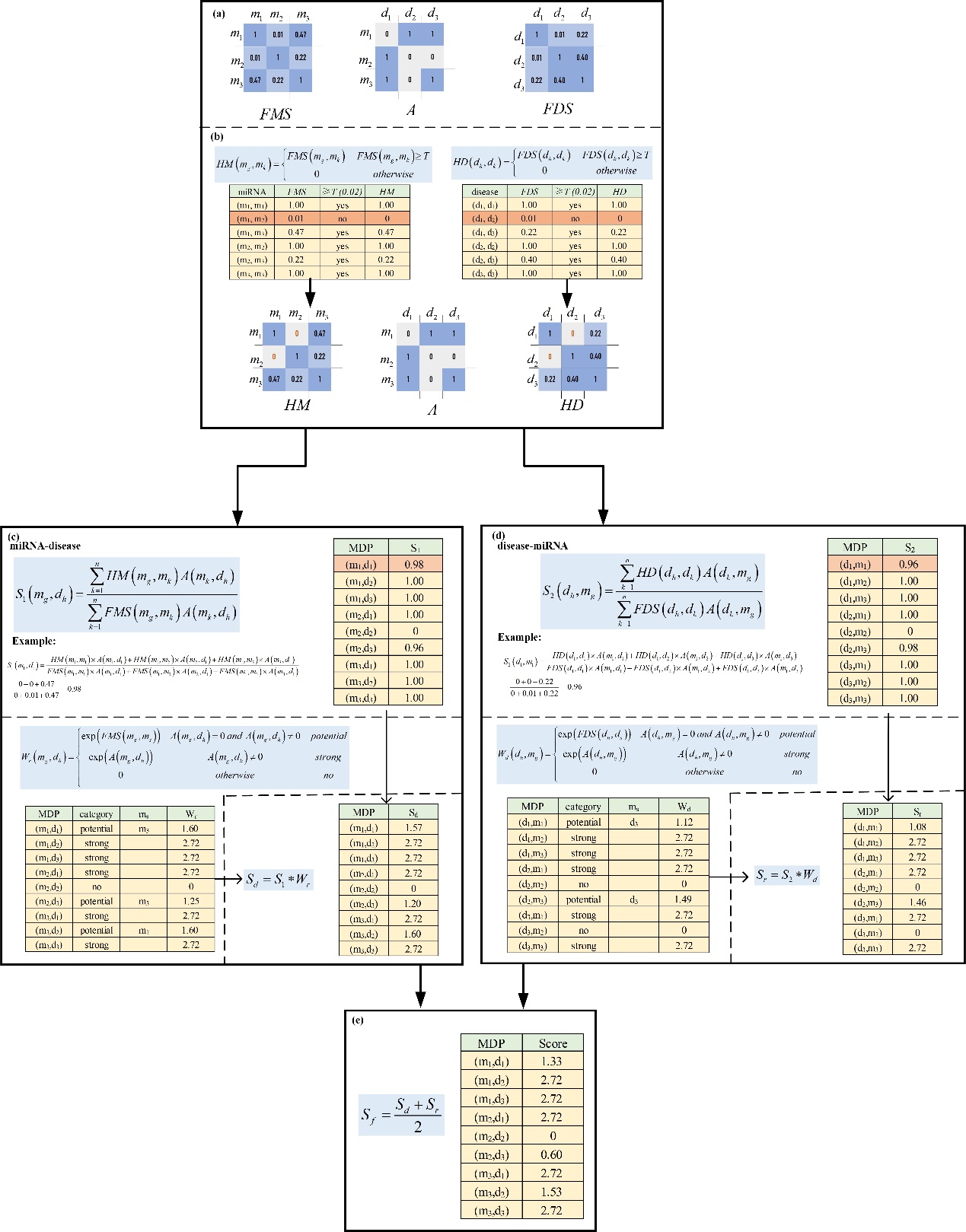


Figure S2. A BLNIMDA calculation example, including the generation of two side information properties, the calculation of two affinity weights for each MDP and the MDA score.
